# A Rapid Detection Method for Tomato Gray Mold Spores in Greenhouse Based on Microfluidic Chip Enrichment and Lens-Less Diffraction Image Processing

**DOI:** 10.3390/foods10123011

**Published:** 2021-12-05

**Authors:** Yafei Wang, Hanping Mao, Xiaodong Zhang, Yong Liu, Xiaoxue Du

**Affiliations:** 1 School of Agricultural Engineering, Jiangsu University, Zhenjiang 212013, China; 2111916018@stmail.ujs.edu.cn (Y.W.); 1000001703@stmail.ujs.edu.cn (X.Z.); 2111816007@stmail.ujs.edu.cn (Y.L.); 2111916004@stmail.ujs.edu.cn (X.D.); 2 Key Laboratory of Modern Agricultural Equipment and Technology, Ministry of Education, Jiangsu University, Zhenjiang 212013, China

**Keywords:** greenhouse, crop airborne disease, lens-free, light diffraction, image processing, microfluidic chip

## Abstract

It is of great significance to find tomato gray mold in time and take corresponding control measures to ensure the production of tomato crops. This study proposed a rapid detection method for spores of *Botrytis cinerea* in green-house based on microfluidic chip enrichment and lens-free diffraction image processing. Microfluidic chip with a regular triangular inner rib structure was designed to achieve the enrichment of *Botrytis cinerea* spores. In order to obtain the diffraction image of the diseased spores, a lens-less diffraction imaging system was built. Furthermore, the collected spore diffraction images were processed and counted. The simulation results showed that the collection efficiency of 16 μm particles was 79%, 100%, and 89% at the inlet flow rate of 12, 14 and 16 mL/min, respectively. The experimental verification results were observed under a microscope. The results showed that when the flow rate of the microfluidic chip was 12, 14 and 16 mL/min, the collection efficiency of *Botrytis cinerea* spores was 70.65%, 87.52% and 77.96%, respectively. The *Botrytis cinerea* spores collected in the experiment were placed under a microscope for manual counting and compared with the automatic counting results based on diffraction image processing. A total of 10 sets of experiments were carried out, with an error range of the experiment was 5.13~8.57%, and the average error of the experiment was 6.42%. The Bland–Altman method was used to analyze two methods based on diffraction image processing and manual counting under a microscope. All points are within the 95% consistency interval. Therefore, this study can provide a basis for the research on the real-time monitoring technology of tomato gray mold spores in the greenhouse.

## 1. Introduction

The area of various greenhouses has been increased up to 2.1 million hectares in China since 2017 [1,2]. Tomato is one of the important widely planted vegetables in the world as well as in China and has high economic and health benefits [3,4]. At present, China has become the world’s largest producer and consumer of tomatoes, with a planting area of 1.01 million hectares [1,5]. Tomato gray mold is caused by the asexual fungus *Botrytis cinerea* (*B. cinerea*). It’s one of the common diseases in greenhouse tomato cultivation [6]. *B. cinerea* can spread through the air from disease spores [7]. *B. cinerea* occurs early and lasts for a long time, mainly at flowering and fruiting stages, flowers, fruits, leaves, and stems [6,8]. Suitable environment conditions in the greenhouse are favorable to the occurrence of *B. cinerea*, and it is not easy to control [9]. Generally, after *B. cinerea* disease occurs, the yield of tomatoes is decreased by 20–30%, and the severe plots can even be as high as about 50% [10,11,12]. Therefore, how to quickly and accurately discover *B. cinereae* and take corresponding control measures is of great significance to ensure the production of tomato crops [4,13].

Timely monitoring of *B. cinerea* spores is the key to effectively controlling its incidence [14]. At present, the diagnosis and prevention of greenhouse crop diseases are based on the experience of producers and the results of routine tests in the laboratory or on-site [5,7]. Laboratory testing techniques mainly include electron microscopy testing technology, polymerase chain reaction (PCR), and biological testing technology, etc. These testing technologies can accurately determine the type of disease, but laboratory testing technology is destructive, time-consuming, and labor-intensive [15,16,17]. Spectral detection technology and image processing technology are used to detect known or specific diseases. These technologies have good detection accuracy for the inversion of diseases through statistical modeling and provide accurate guidance for disease prevention and control [6,9,13]. However, these diagnostic techniques cannot detect crop diseases before they become prevalent but can only be detected when the diseases occur. At this time, the optimal window for prevention and control has been missed [18,19].

Relevant scholars use portable spore traps to catch airborne disease spores and combine them with image processing to detect airborne disease spores of crops in the early stage, e.g., Lei et al. [20], in order to realize the early detection of airborne diseases spores. Urediniospores were collected by using portable spore traps. The urediniospores were automatically detected and counted using a series of image processing approaches, including image segmentation using the K-means clustering algorithm, image pre-processing, the identification of touching urediniospores based on their shape factor and area. Wang et al. [7] achieved the identification and classification of three kinds of airborne disease spores of greenhouse crops. The spores of three airborne diseases were collected by portable spore traps. They proposed a method to identify the spores of greenhouse crop airborne diseases by digital image processing. Collected spore images were pre-processed. Then, the color, shape, and texture characteristics of spores were extracted, and the classification models of the spores were built based on logistic regression (LR), K-nearest neighbor (KNN), random forest (RF), and support vector machine (SVM), respectively. Yang et al. [21] in order to achieve early detection of rice blast proposed a method to detect and identify rice blast based on crop disease spores’ diffraction fingerprint texture, which has certain advantages compared with the existing method of manual identification by microscope. Although the above methods can achieve early detection of crop airborne diseases, they still face many problems. First, the composition of air is complex. There is particulate matter in the air including spores, pollen, PM10, aerosols, etc. The spores of *B. cinerea* disease are challenging to directly separate. Secondly, the concentration of *B. cinerea* spores in the air before the disease outbreak was low, and it was difficult to detect it directly. Finally, the detection of diseased spores requires the help of a microscope, while the traditional optical microscope equipment has a small imaging area. In the early stage of airborne diseases, the spore concentration is low and the detection is difficult. In addition, the traditional optical microscope equipment is large in size and expensive, which cannot meet the needs of the large-area deployment.

In recent years, with the development of microfluidic technology, it is possible to separate and enrich small particles. For example, Yang et al. [22], in order to detect the diseased rice spores, designed a microfluidic chip to enrich and separate the diseased rice spores. Rice disease spores can be collected in the chip enrichment region. Wang et al. [23] designed a microfluidic chip that can directly enrich airborne fungal spores from airflow. The chip includes three parts: half-wave pretreatment channel, inertial impactor, and low-pressure collection chamber. Lee et al. [24] designed a microfluidic device to achieve high separation performance for continuous separation of nanoparticles by combining diffusiophoresis and electrophoresis to achieve the separation of nanoparticles having different sizes. In summary, microfluidic technology can be used to achieve separate and enrich *B. cinerea* spores in the greenhouse.

Hence, in this study, in order to achieve the enrichment of *B. cinerea* spores, a rapid detection method for *B. cinerea* spores in the greenhouse based on microfluidic enrichment and lens-less diffraction image processing designed a microfluidic chip with a regular triangular inner rib structure. Furthermore, built a lens-less diffraction imaging system which can separation-enrichment of greenhouse *B. cinerea* disease spore and achieve timely monitoring.

## 2. Materials and Methods

### 2.1. Spore Sample Preparation

**Figure 1:**
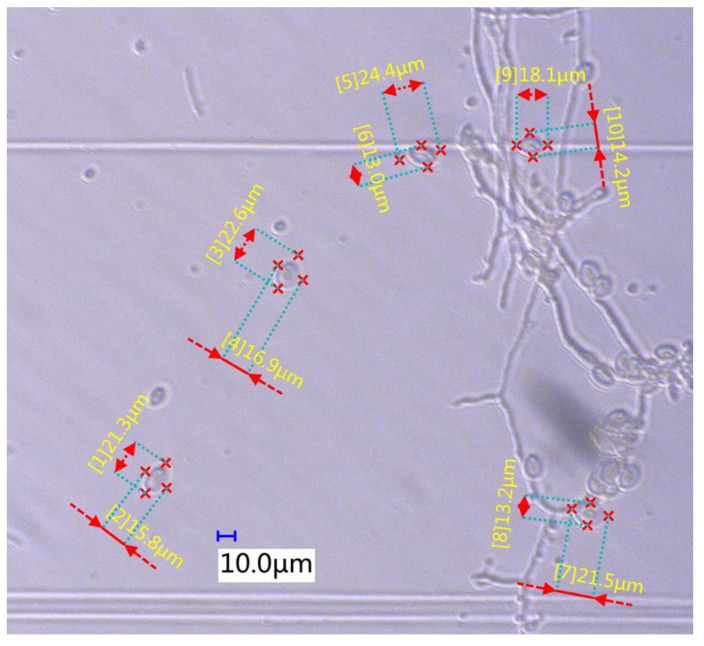
The measurement results for *B. cinerea* spores.

*B. cinerea* spores were collected from tomato leaves. *B. cinerea* spores were maintained on potato dextrose agar medium and maintained on tomato plants by periodic transfer to the new plants when necessary [2,7]. Then, the sporangia or conidia suspension was prepared. Finally, the size parameters of spores were measured by the super depth of field microscope (VHX-900F, made by KEYENCE Co., Osaka, Japan). The measurement results are shown in Figure 1. Statistical analysis of the measured spores showed that *B. cinerea* spores are almost oval and have a size distribution of 19.3 (11.4–26.7) × 11.7 (8.3–14.5) μm^2^.

### 2.2. Design of Microfluidic Chip

**Figure 2:**
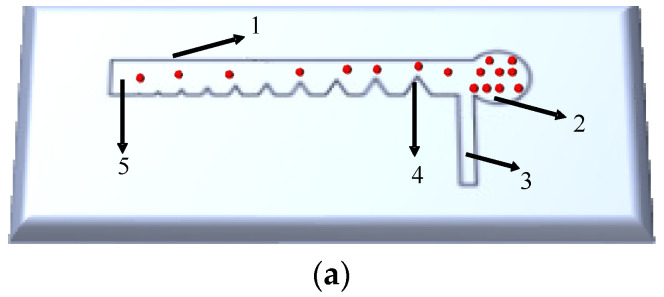

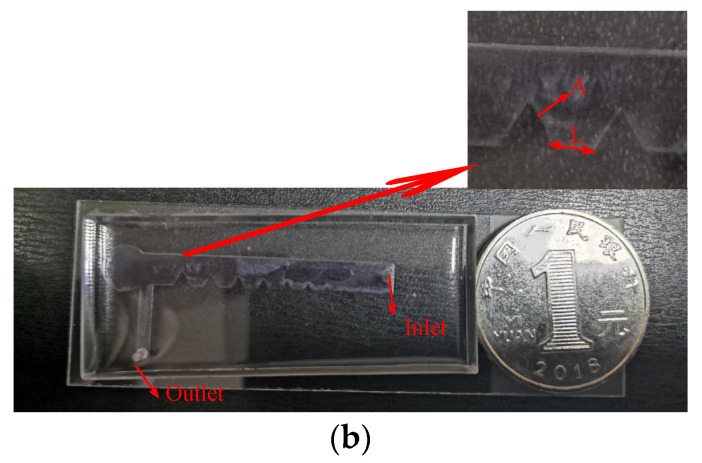
Structure diagram of microfluidic chip: (**a**) Two-dimensional structure,1. Plane structure, 2. Collection tank, 3. Channel 1, 4. Internal rib structure, 5. Channel 2; (**b**) Physical picture of the microfluidic chip, A and L represent the side length of internal rib structure and the internal rib structure spacing, respectively.

In order to achieve efficient separation and enrichment of greenhouse *B. cinerea* spores, a microfluidic chip with a regular triangular inner rib structure for separation and enrichment of the greenhouse *B. cinerea* spores directly from gas flow was developed. The microfluidic chip was prepared according to the method mentioned in reference [22,25,26]. Figure 2 is the structure diagram of the microfluidic chip, which composed of plane structure, collection tank, internal rib structure, Channel 1, and Channel 2. The internal rib structure is a regular triangle, and the side length is A, the spacing of the internal rib structure is L, and the radius of the collection tank is R.

### 2.3. Numerical Method

In this study, the finite element analysis software COMSOL Multiphysics 5.1 was used for simulation analysis. Using the particle tracking module in COMSOL, the particle trajectories can be predicted. For numerical analysis, the model parameters were set at width of channel 1 = 1500 µm, length of channel 1 = 10,000 µm, width of channel 2 = 4000 µm, width of channel 2 = 33,750 µm. The internal rib structure is a regular triangle, and the side length is A (from 250 µm to 2500 µm, and the interval is set to 250 µm). The spacing of the internal rib structure is L (from 500 µm to 1500 µm, and the interval is set to 500 µm). The radius of the collection tank is R (from 2500 µm to 5000 µm, and the interval is set to 500 µm).

In a microfluidic system, laminar flow corresponds to the flow with small Re (Re < 1), the viscosity term of the Navier–Stokes equation dominates, and the inertia term can be ignored. At this point, the corresponding boundary conditions are: (1) the channel wall is a non-slip boundary. (2) The flow rates of inlet was set at 12, 14, and 16 mL/min, respectively. (3) The pressure at the outlet is P = 0. The flow was assumed to be steady, two-dimensional axisymmetric, and incompressible. The particle density was set at 1050 kg/m^3^ to express the aerodynamic size. Spore concentration in the air is deficient, so 100 particles were released from the particle entrance. Adjusting the convergence criterion to 10^−6^, iteratively solve the continuity, momentum, and energy equations. The Lagrange method is used to represent particle motion. The equation of motion of the particle is expressed as follows, Equation (1):

(1) $$m_{P}\frac{d^{2}r_{P}}{dt^{2}}=F_{f}+F_{O}$$

where $r_{p}$ is used to represent the particle motion position vector, $m_{p}$ is used to represent the particle mass, and t is used to represent the particle motion time. $F_{f}$ is used to represent the force of fluid on particles, and $F_{O}$ is used to represent the force exerted by the external potential field on particles.

The motion behavior of particles was investigated based on flow analysis. Using post-processing in COMSOL, select the global calculation in the derived value. The data set selects the particles to be released, and the expression is the total number of particles in the selection. Count the particles collected in the collection tank area. The enrichment efficiency of particles was obtained.

### 2.4. Diffraction Image Detection Platform Setup

**Figure 3:**
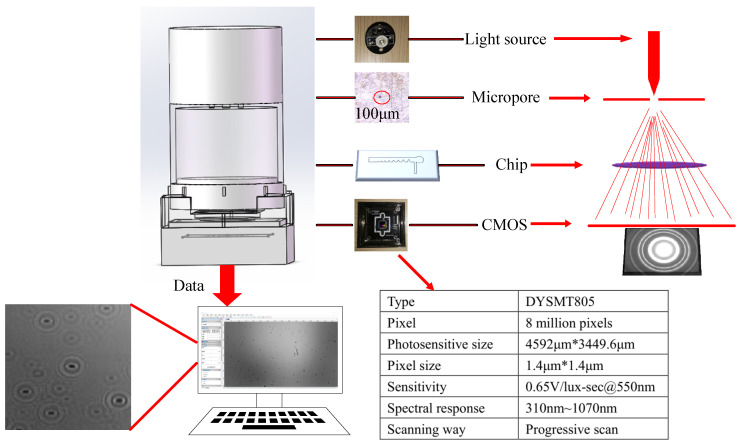
Diffraction imaging detection platform.

Traditional micro-optical imaging technology refers to that visible light can be transmitted through or reflected from the sample, and after passing through one or more lenses, the magnified image of the tiny sample can be obtained. Diffraction is a phenomenon in which light waves deviate from the straight path and travel behind obstacles when they encounter obstacles or small holes during the propagation process. Observe the bright and dark areas that appear on the screen. The light intensity distribution can reflect the imaging information of the object. The diffraction imaging detection platform is shown in Figure 3.

The facility has two main parts—the microfluidic chip and the lens-less imaging module (includes the light source, stoma, and CMOS sensor) and the light source is LED light (520~525 nm). The diameter of the pores is 100 µm directly below LED light. and the CMOS sensor is located 45 mm below the micropore. The DYSMT805 image sensor chip (Changsha Daying Electronic Technology Co., Ltd., Changsha, China) with 8 million pixels was selected. Imaging area is 4592 µm × 4339.6 µm. The pixel size is 1.4 µm ×1.4 µm. The sensitivity of CMOS sensor is 0.65 V/lux-sec@550 nm. The operating temperature range is 0 ~ 50 °C. The spectral response is 310 nm ~ 1070 nm. Due to the LED light source being monochromatic light, and the CMOS sensor collects color images. The image mode was set to gray in the image acquisition software (ToupView, Suzhou Jingtong Instrument Co., Ltd., Suzhou, China). The facility realizes the independent operation of spore enrichment, sampling, photographing and detection.

The diffraction imaging system in this research is designed according to the Huygens–Fresnel principle. Diffraction images satisfy the relationship between Fourier transform and inverse transform. The complex amplitude of point P on the diffraction image can be expressed as Equation (2).

(2) $$U\left(P\right)=k\iint^{\text{​}}U_{0}\left(Q\right)F\left(\theta_{0},\theta \right)\frac{r^{ikr}}{r}dxdy=\left|U\left(P\right)\right|\cdot e^{iap}$$

where |*U*(*P*)|is used to represent amplitude information; *e^iaq^* is used to represent phase information. CMOS sensor is used to take diffraction images of airborne disease spores. The square of the light intensity information amplitude of the diffraction image is Equation (3):

(3) $$I\left(P\right)={\left|U\left(P\right)\right|}^{2}$$

Due to the inevitable phase loss phenomenon in the diffraction imaging process, it is necessary to perform phase recovery processing on the diffraction image of airborne spores. In this study, sampling theory and iterative algorithms are used to recover the phase of airborne disease spores diffraction images.

### 2.5. Diffraction Image Processing and Counting

**Figure 4:**
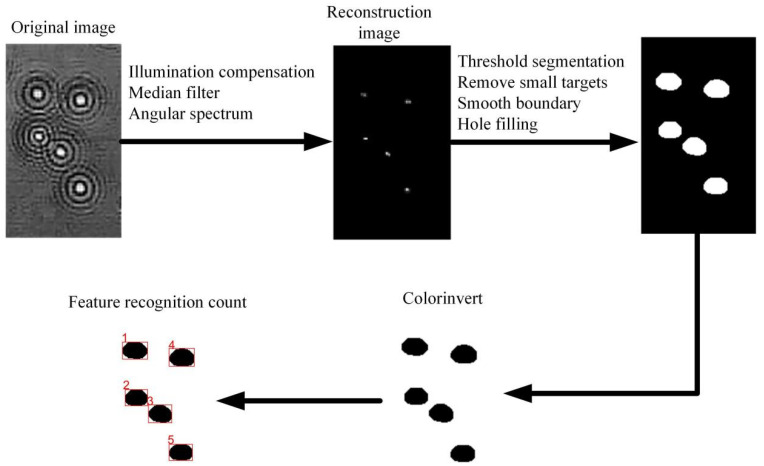
Spore diffraction image processing and counting.

The diffraction image processing process of airborne disease spores includes two parts: diffraction image pre-processing and feature information extraction. Diffraction image pre-processing reduces useless information, which is beneficial to obtain effective information. The flow chart of diffraction image processing and counting is shown in Figure 4.

Firstly, two-dimensional gamma function is used to correct the spore diffraction image of airborne diseases. The expression for the gamma function is, Equations (4) and (5).

(4) $$O\left(x,y\right)=255{\left(\frac{F\left(x,y\right)}{255}\right)}^{\gamma }$$

(5) $$\gamma ={\left(\frac{1}{2}\right)}^{\frac{m-I\left(x,y\right)}{m}}$$

where *O*(*x*, *y*) is used to represent two-dimensional gamma functions, *F*(*x*, *y*) is used to represent the source image, *I*(*x*, *y*) is used to represent the light component, *m* is used to represent average brightness.

Secondly, in order to reduce the salt and pepper noise in the spore diffraction image of airborne disease, a median filter was used. Reconstruction of diffraction image of airborne disease spores by combined angular spectrum reconstruction algorithm. The formula of angular reconstruction is as follows, Equation (6):

(6) $$U\left(x_{i},y_{i},z_{i}\right)=F^{-1}\left\{F\left[C\left(x,y\right)\cdot H\left(x,y\right)\right]G_{AS}\left(f_{x},f_{y}\right)\right\}$$

where *U*(*x_i_*, *y_i_*, *z_i_*) is used to represent the complex amplitude distribution of light, *C*(*x*, *y*) is used to represent reproduction lightwave, *H*(*x*, *y*) is used to represent the intensity distribution of hologram, *G*_AS_(*f_x_*, *f_y_*) is the free space transfer function.

Fourthly, the reconstructed image is finalized, and block threshold segmentation is used to filter out useless information. Fifthly, filling holes in the final image. Sixthly, after the holes are filled, smooth the image boundary. Seventhly, holes in large areas being filled. Eighthly, extract the two morphological features of airborne disease spore area and roundness, and identify and count them. The formula is as follows, Equations (7) and (8):

*A* = *N* (7)

(8) $$P=\sqrt{2}N_{o}+N_{e}$$

where *A* is used to represent the area of the processed spore image, *N* is used to represent the number of pixels of the processed spore image, *P* is used to represent the perimeter of the processed spore image, *N_o_* is used to represent the number of diagonal pixels of the processed spore image, *N_e_* is used to represent the number of horizontal or vertical pixels in the processed spore image.

### 2.6. Statistical Analysis

The data were analyzed using the statistical analysis software program SPSS. The statistical differences between groups were analyzed by using ANOVA. The least significant difference (LSD) test was used to determine a significance level of *p* < 0.05. The Bland–Altman method was used to analyze two methods based on diffraction image processing counting and manual counting under a microscope.

## 3. Results and Discussion

### 3.1. Particle Motion Simulation

**Figure 5:**
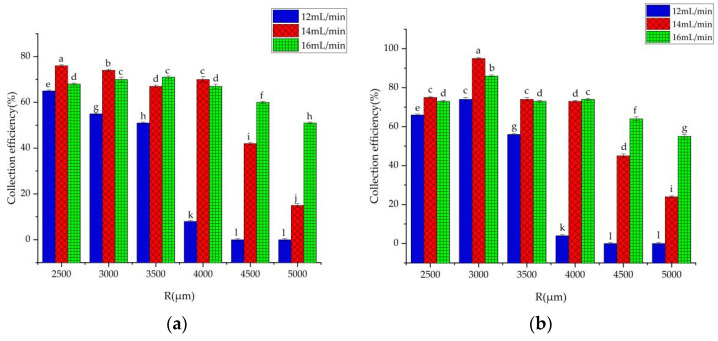

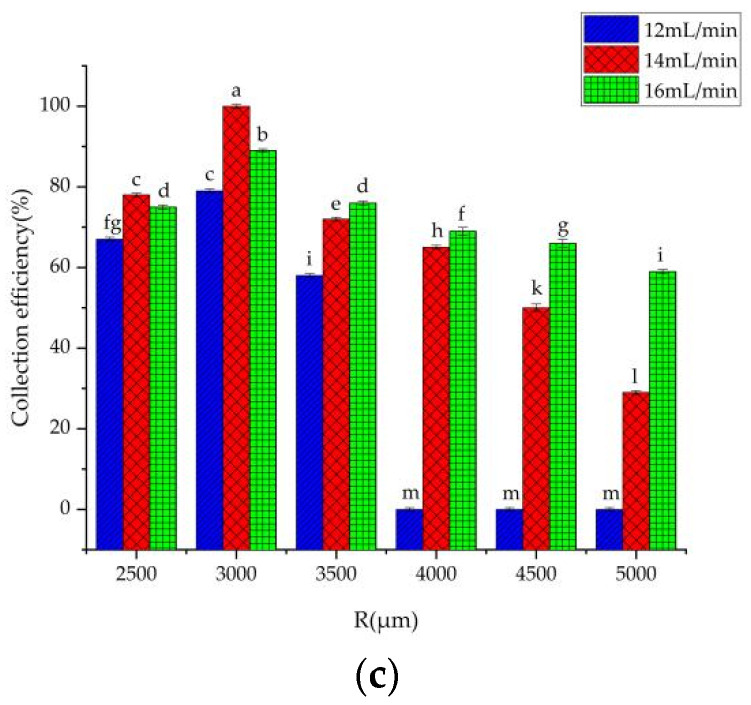
Collection efficiency of 16 μm particles. (**a**) Spacing of the internal rib structure L = 500 μm, (**b**) Spacing of the internal rib structure L = 1000 μm, (**c**) Spacing of the internal rib structure L = 1500 μm, a–m are used to represent significance levels.

The spores in the air are mainly ungerminated [27]. In this study, 16 µm particles were used to represent *B. cinerea* spores during the simulations. In this section, the internal rib structure is a regular triangle, and the side length is A (from 250 µm to 2500 µm, and the interval is set to 250 µm). In order to find the optimal spacing of the internal rib structure, different values (from 500 µm to 1500 µm, and the interval was set to 500 µm) of the spacing of the internal rib structure were used to simulate. In order to seek the optimal radius of the collection tank, different values (from 2500 µm to 5000 µm, and the interval is set to 500 µm) of the collection tank’s radius were used to simulate. The enrichment results of particles are shown in Figure 5.

**Figure 6:**
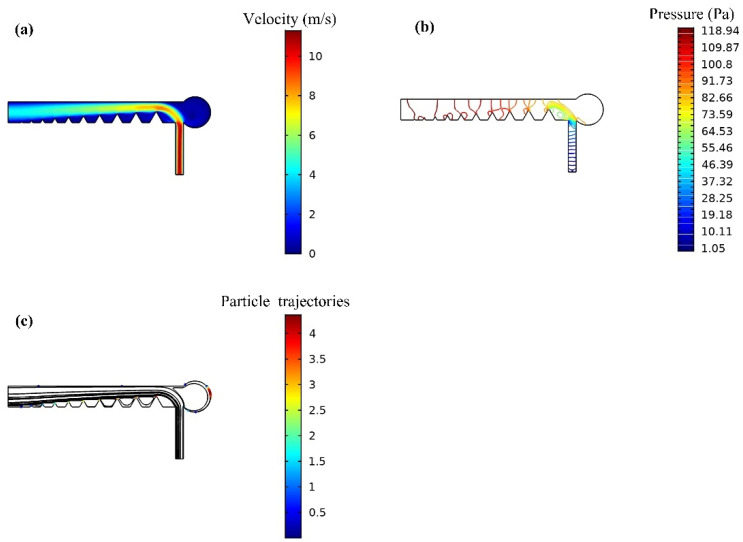
Simulation results of the microfluidic chip. (**a**) Velocity distribution, (**b**) Pressure distribution, (**c**) 16 μm particle trajectory.

It can be seen from Figure 5a that when L = 500 μm, the collection efficiency of 16 μm particles decreases with the increase of the radius R of the collecting tank. However, the difference is that in Figure 5b,c, when L = 1000 μm and L = 1500 μm, the collection efficiency of particles increases first and then decreases with the increase of the radius R of the collecting tank reaches its maximum when the radius R of the collecting tank is 3000 μm. Combined with the influence of the inlet flow rate of the microfluidic chip on the collection efficiency of 16 μm particles, when R = 3000 μm, L = 1500 μm, the collection efficiency of 16 μm particles is relatively high. The maximum collection efficiency of 16 μm particles was 79%, 100% and 89% at the inlet flow rate of 12, 14 and 16 mL/min, respectively. Therefore, R = 3000 μm, L = 1500 μm, and flow rate of 14 mL/min were set as the next step of numerical simulation in this study. Figure 6a is the velocity distribution in the microchannel of the chip, Figure 6b is the intensity of pressure distribution in the microfluidics of the chip, and Figure 6c is the simulation results of 16 μm particles.

As shown in Figure 6, the airflow enters the microchannel from the particle’s entrance and obtains a horizontal to right initial velocity [28]. The particles move forward under the action of airflow, but in the process of the particles moving forward, due to the triangular inner rib structure, the direction of the particle movement will change. The particles suspended in the airflow are injected into the traditional inertial separation system, and some particles with sufficient momentum can pass through the streamline and be separated. However, other airflows with insufficient momentum will leave as the airflow deflected [29]. It can be seen from Figure 6a,b that the velocity of particles in the collecting tank is close to 0, and there is no pressure distribution in the collecting tank. It can be seen from Figure 6c that 16 μm particles can be well collected in the collecting tank. It is proved that the designed microfluidic chip can be used to collect 16 μm particles.

### 3.2. Spore Collection Experiment

#### 3.2.1. Evaluation of *B. cinerea* Spores Collection Efficiency

The collection efficiency of *B. cinerea* spores can be defined as Equation (9) [30].

(9) $$\eta =\frac{\eta_{i}}{\eta_{i}+\eta_{j}}\times 100\%$$

where $\eta_{i}$ is the number of given particle size spores that pass through the chip and enter the collection tank; $\eta_{j}$ is the sum of the number of given particle size spores at the microchannel wall and outlet position of the chip.

#### 3.2.2. Result of Spore Collection

**Figure 7:**
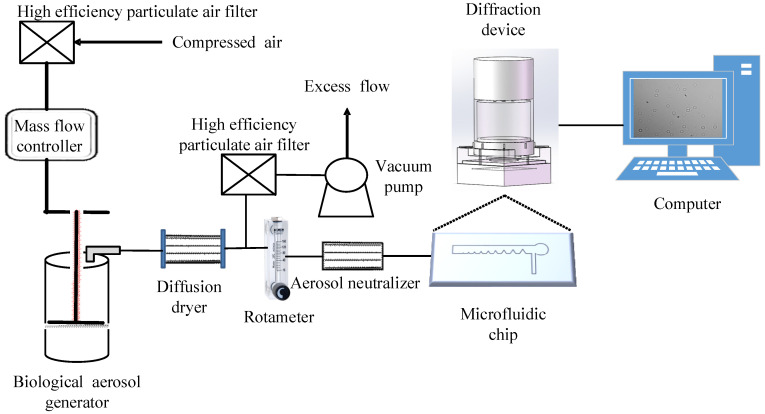
Schematic diagram of the experimental platform.

The schematic diagram of the experimental platform is shown in Figure 7, including high-efficiency particulate air filter, mass flow controller, biological aerosol generator, diffusion dryer, rotameter, aerosol neutralizer, vacuum pump, microfluidic chip, diffraction device, and computer system.

*B. cinerea* spores suspension from greenhouse crops was placed in aerosol generators to maintain the integrity and biological activity of spores under low pressure. The compressed air was filtered with high-efficiency particulate air to produce a bioaerosol at 2 atmospheres. A diffusion dryer was placed behind the aerosol generator (24 Jet Collison, BGI Collison) to remove moisture from the aerosol stream. A ^210^Po aerosol neutralizer was installed after the diffusion dryer to remove the charge from the spores [5,23]. The flow rate of the rotameter (range of rotameter is 6–60 mL/min) was set to 12, 14 and 16 mL/min, respectively. Finally, the PDMS layer was removed to expose spores, so the collected greenhouse crops’ airborne disease spores can be observed by the inverted microscope.

**Figure 8:**
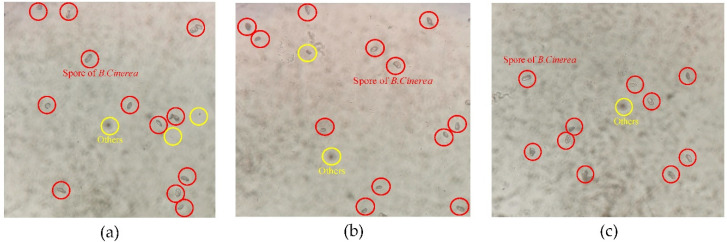
Experimental results of spores enrichment. (**a**) Example 1, (**b**) Example 2, (**c**) Example 3.

Figure 8 is an experimental image taken by the inverted microscope. As can be seen from Figure 8, the spores of *B. cinerea* can be well collected in the enrichment area. In addition, the distribution of spores in the enrichment area is relatively scattered, and there is little overlap. This is conducive to the subsequent collection and processing of spore diffraction images. However, by analyzing the collected spore microscopic images, it can be known that there are still some other impurities in the collection tank of the microfluidic chip. This may be because the air has some impurities similar in volume and density to tomato gray mold spores. Therefore, the designed microfluidic chip can separate and collect tomato gray mold spores.

In this study, to verify the collection efficiency of *B. cinerea* spores, the designed microfluidic chip was tested. A total of three sets of experiments were performed, and the flow rates of the three sets of experiments were set to 12, 14 and 16 mL/min, respectively. To reduce the random error of the experiment, experiments were repeated five times, each experiment was conducted for 2 min, and the average value and standard deviation were calculated at last. Then, the experimental results were statistically analyzed. The result is shown in Table 1.

It can be seen from Table 1 that when the flow rate of the microfluidic chip was 12, 14, and 16 mL/min, the collection efficiency of *B. cinerea* spores was 70.65%, 87.52% and 77.96%, respectively. The standard deviation analysis of the five tests showed that when the flow rate of the microfluidic chip was 12, 14, and 16 mL/min, the standard deviation of the test results was 0.024, 0.012 and 0.025, respectively. The collection efficiency was the best when the flow rate of the microfluidic chip was 14 mL/min. The experimental results are consistent with the simulation results. It can be seen from Figure 5 that when R = 3000 μm, L = 1500 μm and the flow rates was 12, 14 and 16 mL/min, respectively, the maximum collection efficiency of the designed microfluidic chip for 16 μm particles was 79%, 100% and 89%, respectively. The actual collection efficiency of the microfluidic chip for *B. cinerea* spores was slightly lower than the simulation result. This may be because the model of *B. cinerea* spores was an equivalent model during the simulation, while the size of *B. cinerea* spores in the actual test was within a range (in Figure 1). In addition, it can be seen from Table 1 that the deviations of the three sets of tests are all within the range of ±5%, which can meet the actual engineering needs. Therefore, the microfluidic chip can realize the enrichment of *B. cinerea* spores in the greenhouse and provide a basis for the study of real-time monitoring technology of *B. cinerea* spores in the greenhouse.

### 3.3. Spore Count Results

#### 3.3.1. Evaluation of *B. cinerea* Spores Count Results

The counting error of *B. cinerea* spores can be defined as, Equation (10).

(10) $$error=\frac{\left|N-N_{0}\right|}{N_{0}}\times 100\%$$

where *N* is the counting result of computer image processing. *N*_0_ is the result of manual counting under a microscope.

#### 3.3.2. Analysis of *B. cinerea* Spores Count Results

The *B. cinerea* spores collected in the experiment were placed under a microscope for manual counting and compared with the automatic counting results based on diffraction image processing. The results are shown in Table 2.

It can be seen from Table 2 that a total of 10 sets of experiments were carried out, with an error range of the experiment was 5.13~8.57%, and the average error of the experiment was 6.42%. Although the automatic counting based on diffraction image processing has a certain error compared with manual counting under the microscope, the overall accuracy is still trustworthy and falls within the acceptable range. The source of error may be that some diffraction rings are weak and difficult to identify when using diffracted light. It is also possible that the suspension of *B. cinerea* spores used in the experiment contained spores at different germination stages, and the shapes and sizes of spores at different germination stages were different, resulting in different diffraction fingerprints. In actual conditions, the spores in the air are in the ungerminated stage [27]. Only when the *B. cinerea* spores invade tomato leaves and fruitscan germinate under a suitable temperature and humidity environment.

**Figure 9:**
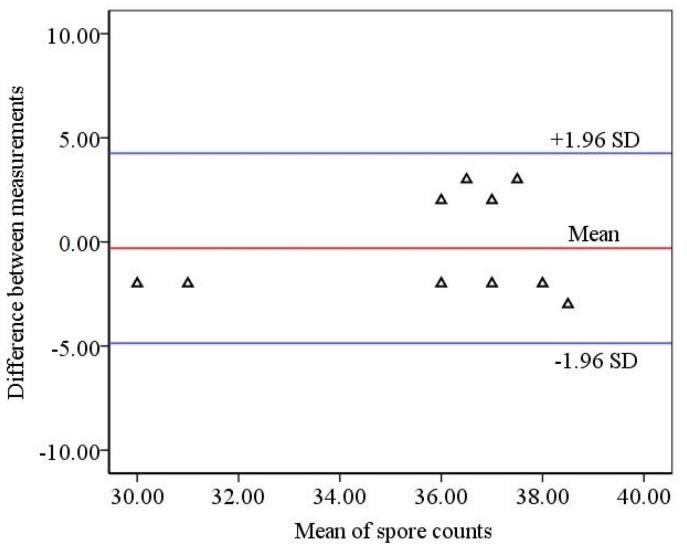
Analysis by Bland–Altman method, the triangles in the figure represent data for counting errors.

In order to further illustrate the feasibility of identifying and counting spores of *B. cinerea* based on diffraction image processing, the Bland–Altman method was used to analyze two methods based on diffraction image processing counting and manual counting under a microscope. The analysis results are shown in Figure 9. It can be seen from Figure 9 that all points are within the 95% consistency interval, and the consistency is good [31]. The results showed that the recognition and counting results of *B. cinerea* spores based on diffraction image processing had good consistency with the manual counting results, and it could be used for the identification and counting of *B. cinerea* spores.

## 4. Conclusions

Tomatoes are amongst the most important and widely planted vegetables in the world as well as in China and has high economic and health benefits. Generally, after tomato gray mold fungi occurs, the yield of tomato is decreased by 20–30%, and the serious plots can even be as high as about 50%. Therefore, how to quickly and accurately discover *B. cinereae* and take corresponding control measures is of great significance to ensure the production of tomato crops. In this study, in order to realize early monitoring of tomato gray mold, a rapid detection method for spores of *B. cinerea* in a greenhouse was proposed based on microfluidic chip enrichment and lens-free diffraction image processing and designed a microfluidic chip with a regular triangular inner rib structure. Built a lens-less diffraction imaging system. In order to verify the collection efficiency of *B. cinerea* spores, the designed microfluidic chip was tested. When R = 3000 μm, L = 1500 μm, and the flow rate was 14 mL/min and the collection efficiency of *B. cinerea* spores was 87.52%, the standard deviation of the test results was 0.012. Collection efficiency is the best.

## Figures and Tables

**Table 1:** Statistics results of spore enrichment.

Velocity	Count Type	Experimental Groups						
		G 1	G 2	G 3	G 4	G 5	Average	Standard Deviation
12 mL/min	Collection Tank	82	89	91	87	94	88.6	4.03
	Other areas	28	35	40	38	43	36.8	5.11
	Sum	110	124	131	125	137	125.4	9.00
	Collection Efficiency	74.55%	71.77%	67.91%	69.6%	68.61%	70.65%	0.024
14 mL/min	Collection Tank	94	98	103	87	109	98.2	7.52
	Other areas	12	16	14	11	17	14	2.28
	Sum	106	114	117	98	126	112.2	9.55
	Collection Efficiency	88.68%	85.96%	88.03%	88.78%	86.51%	87.52%	0.012
16 mL/min	Collection Tank	86	92	82	78	84	84.4	4.63
	Other areas	25	32	20	18	23	23.6	4.84
	Sum	111	124	102	96	107	108	9.44
	Collection Efficiency	77.48%	74.19%	80.39%	81.25%	78.5%	77.96%	0.025

Note: G 1–G 5 are the experimental groups.

**Table 2:** Results of manual counting and diffraction image reconstruction recognition counting in the microscope field.

Number	Computer Image Processing Counting	Manual Microscope Counting	Counting Error (%)	Average Counting Error (%)
1	36	38	5.26	6.42
2	39	36	8.33	
3	37	40	7.5	
4	35	37	5.41	
5	37	35	5.71	
6	29	31	6.45	
7	38	35	8.57	
8	37	39	5.13	
9	30	32	6.25	
10	38	36	5.56	
